# Paget–Schroetter syndrome occurring in the nondominant hand

**DOI:** 10.1002/ccr3.8308

**Published:** 2023-12-15

**Authors:** Akina Fukushima, Takaaki Kobayashi, Hayato Kuno, Jiro Hiroki, Akihito Yoshida

**Affiliations:** ^1^ Division of Infectious Diseases Japanese Red Cross Hospital Maebashi Maebashi Japan; ^2^ Division of Infectious Diseases, Department of Internal Medicine University of Iowa Hospitals and Clinics Iowa City Iowa USA; ^3^ Department of Orthopedic surgery Kameda Medical Center Kamogawa Japan; ^4^ Department of Cardiology Kameda Medical Center Kamogawa Japan; ^5^ Department of Internal Medicine Kameda Medical Center Kamogawa Japan

**Keywords:** deep vein thrombosis, Paget–Schroetter syndrome, venous thoracic outlet syndrome

## Abstract

Paget‐Schroetter syndrome is the primary thrombotic event associated with venous thoracic outlet syndrome. It needs to be suspected when encountering localized brachial swelling and a dilated vein in patients with a history of upper limb exercise.

## Graphical abstract

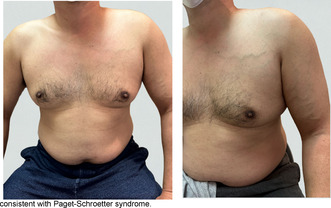

## 1 CASE ILLUSTRATED

**FIGURE 1:**
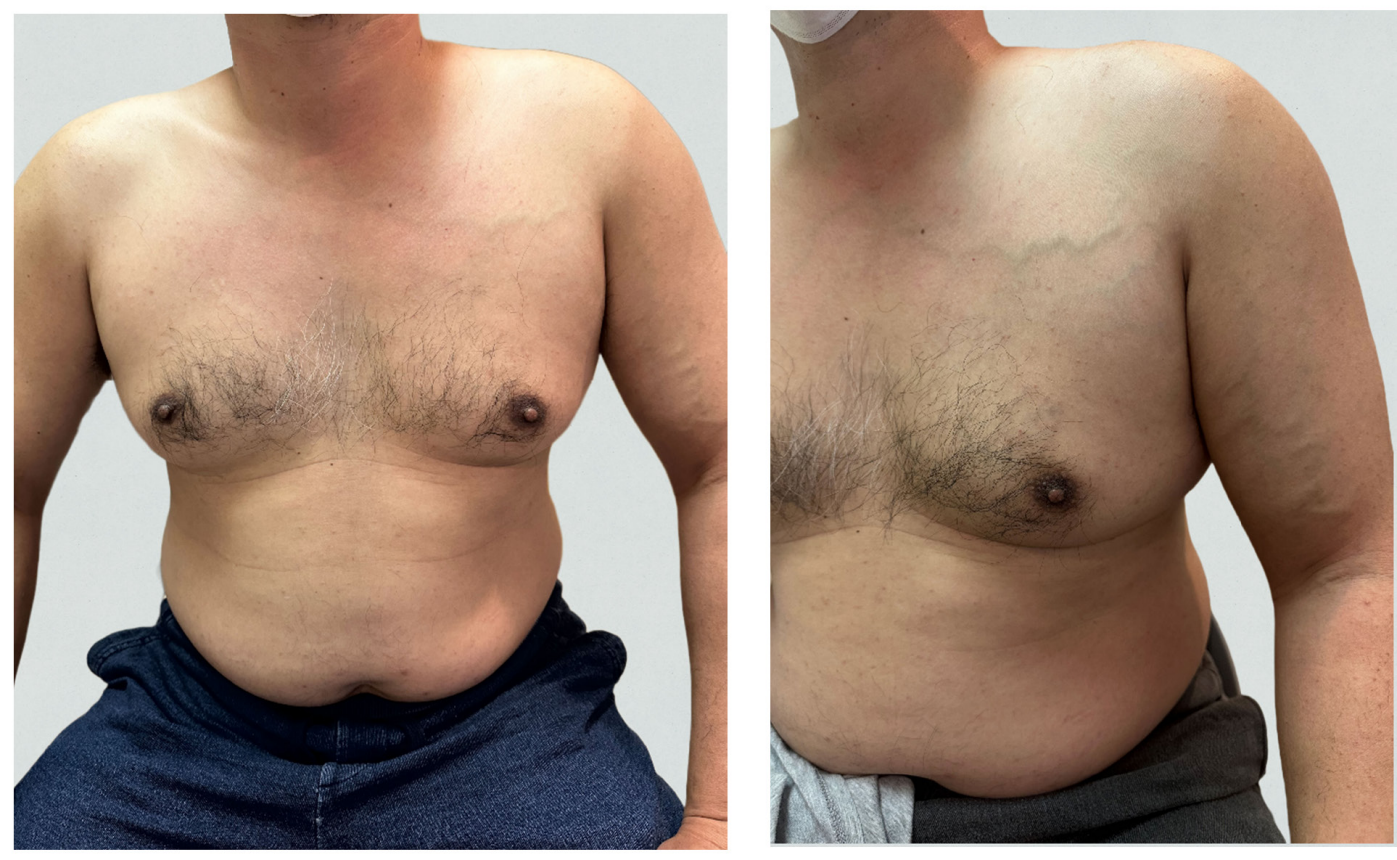
Physician examination revealed left upper limb swelling and vein varicose consistent with Paget–Schroetter syndrome.

**FIGURE 2:**
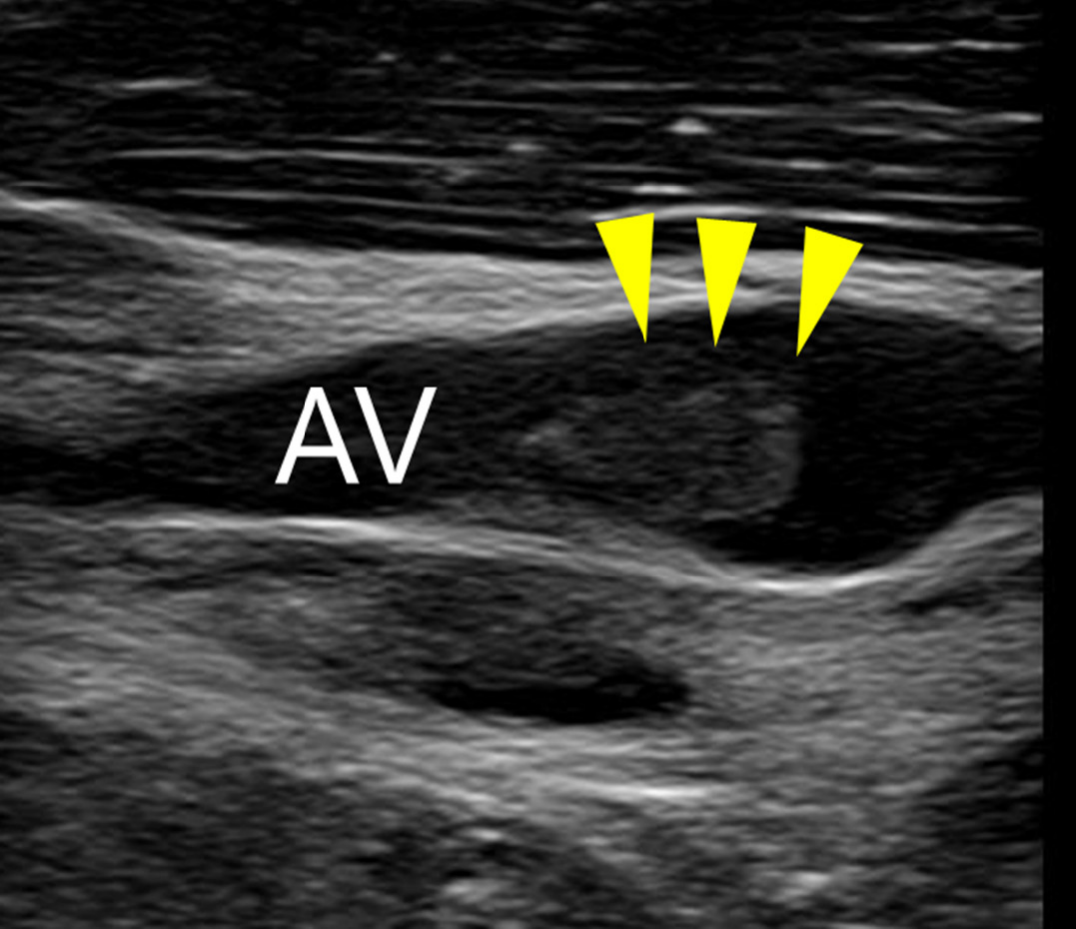
Venous ultrasound showed a 5 mm thrombus in the left axillary vein.

**FIGURE 3:**
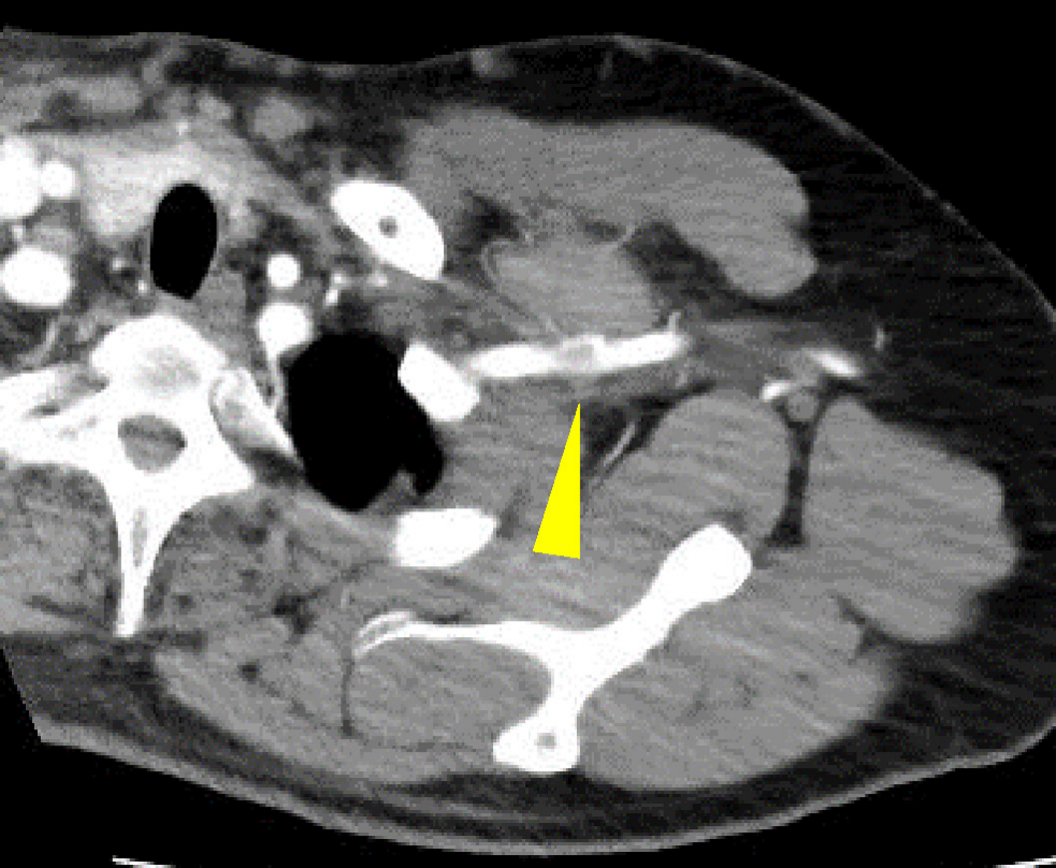
Computed tomography with contrast showed a poorly contrasted area in the superior thoracic aperture indicating Paget–Schroetter syndrome.

A 45‐year‐old man with no significant past medical history presented to our hospital with left shoulder swelling. He is right‐handed and used to participate in baseball during his youth. He has experienced limited extension of his left first to third fingers for the past 8 years. One month before this visit, he developed left shoulder and neck pain. Physical examination showed left neck swelling and a dilated upper limb vein (Figure 1). Laboratory tests, including autoantibody screening and coagulation assessments, yielded unremarkable results, and there were no identified thrombophilic risk factors. Venous ultrasound showed a 5 mm thrombus in the left axillary vein (Figure 2). Contrast‐enhanced computed tomography (CT) of the left upper limbs revealed thromboembolism between the left axillary vein and the left subclavian vein (Figure 3). Venography of the left upper limb showed a thrombosis in the axillary vein below the pectoralis minor muscle. From these findings, the patient was diagnosed with Paget–Schroetter syndrome attributed to thoracic outlet syndrome. We initiated rivaroxaban treatment without improvement after a month. Given the presence of neurological symptoms in the left upper limbs and persistent thrombosis, we opted for surgical intervention. A first rib resection result in complete resolution of his symptoms. We tentatively plan to continue anticoagulation therapy for a total of 6 months and then repeat the CT.

Paget–Schroetter syndrome, also known as effort thrombosis, refers to the primary thrombosis of the subclavian vein at the costoclavicular junction. Thoracic outlet syndrome encompasses three distinct entities: neurogenic (NTOS), venous (VTOS), and arterial (ATOS). VTOS‐associated thrombosis can be categorized into primary and secondary thrombosis. Primary thrombosis occurs spontaneously, whereas secondary thrombosis is linked to catheter insertion or dialysis procedures. Paget–Schroetter syndrome represents the primary thrombotic form of VTOS. The annual incidence of Paget–Schroetter syndrome is estimated to be around 2.03 cases per 100,000 individuals. It predominantly affects men in their thirties, with a higher prevalence in the dominant right hand. Typical symptoms of deep vein thrombosis of the upper limbs are sudden onset, heaviness, pain with swelling, and red‐blue discoloration. If the obstruction is chronic, dilated veins can be seen in the upper arm, base of the neck, and anterior chest wall. Interesting, in our case, nondominant hand was affected. Approximately 60%–80% of patients with Paget–Schroetter syndrome have a history of upper extremity exercise or activities.^1^ Notably, while anticoagulant monotherapy is typically recommended for cases of upper extremity deep vein thrombosis,^2^ this approach alone does not effectively prevent pulmonary embolization in Paget–Schroetter syndrome given about one‐third of patients without corrected anatomical factors experience recurrent clots. Thus, the standard approach involves a combination of catheter‐based thrombolysis and/or surgical intervention (e.g., first rib resection, bypass reconstruction, and patch angioplasty) and anticoagulant treatment. While many previously reported cases required angioplasty or bypass graft, complete recovery was achieved with first rib resection and anticoagulant therapy in the present case.^1^, ^3^ Primary care physicians should consider Paget–Schroetter syndrome when encountering localized brachial swelling and a dilated vein in individuals with a history of intense upper limb exercise. Timely assessment of thrombosis and prompt surgical intervention along with anticoagulation are crucial for optimal management.

## AUTHOR CONTRIBUTIONS

**Akina Fukushima:** Writing – original draft. **Takaaki Kobayashi:** Writing – review and editing. **Hayato Kuno:** Writing – review and editing. **Jiro Hiroki:** Writing – review and editing. **Akihito Yoshida:** Writing – review and editing.

## FUNDING INFORMATION

None.

## CONFLICT OF INTEREST STATEMENT

No disclosure.

## ETHICS STATEMENT

The local ethical committee approval does not apply in this case.

## CONSENT

Written informed consent was obtained from the patient to publish this report in accordance with the journal's patient consent policy.

## Data Availability

Data sharing not applicable to this article as no datasets were generated or analyzed during this study.
